# The Effect of Rye-Based Foods on Postprandial Plasma Insulin Concentration: The Rye Factor

**DOI:** 10.3389/fnut.2022.868938

**Published:** 2022-06-09

**Authors:** Kia Nøhr Iversen, Karin Jonsson, Rikard Landberg

**Affiliations:** Department of Biology and Biological Engineering, Division of Food and Nutrition Science, Chalmers University of Technology, Gothenburg, Sweden

**Keywords:** rye, cereals, structure, insulin, glucose, diabetes, rye factor

## Abstract

Consumption of whole grain has been associated with lower incidence of type-2 diabetes, cardiovascular disease and their risk factors including improved glycemic control. In comparison with other whole grain products, rye bread has been shown to induce lower insulin response in the postprandial phase, without affecting the glucose response. This phenomenon has been referred to as the “rye factor” and is being explored in this review where we summarize the findings from meal and extended meal studies including rye-based foods. Overall, results from intervention studies showed that rye-based foods vs. (wheat) control foods had positive effect on both insulin and glucose responses in the postprandial phase, rather than on insulin alone. Mechanistic studies have shown that the rye factor phenomenon might be due to slowing of the glucose uptake in the intestine. However, this has also been shown for wheat-based bread and is likely an effect of structural properties of the investigated foods rather than the rye *per se*. More carefully controlled studies where standardized structural properties of different cereals are linked to the postprandial response are needed to further elucidate the underlying mechanisms and determinants for the effect of specific cereals and product traits on postprandial glycemic control.

## Introduction

The link between cereal consumption, health and disease has been widely studied and whole grain intake has been consistently associated with lower risk of developing or dying from several major non-communicable diseases such as cardiovascular disease, type-2 diabetes, colorectal cancer, and their main risk factors (1–5). On the contrary, refined grains have been associated with increased or no difference in risk of similar conditions (6, 7). Moreover, there is a general consensus that food grain structure is of importance primarily for blood glucose response, but also risk factors such as cholesterol and low-grade inflammation (8–11), where coarser whole grain cereals have been shown to have positive health benefits, while cereals of more refined character have been shown to have a negative impact on health parameters (3, 12, 13). Authorities in several countries recommend consumption of whole grains instead of refined grains (14, 15). However, some studies have indicated that the effect of cereal consumption may vary among different types of cereals, which could be attributed to variations in content of dietary fiber, bioactive components or other features (16–21). For example, some studies have indicated that glycemic control is improved when the whole grain intake is dominated by rye (6, 19, 22). Rye has the highest dietary fiber content among the cereals and it typically reaches 20% of dry matter and beyond, whereas wheat, oat and barley have approximately 10–15%, although the fiber content differ somewhat depending on variety and cultivation (18, 23). Furthermore, the dietary fiber content of commercially available cereal products may also differ widely, depending on formulation and processing (24, 25). Nonetheless, rye has higher total dietary fiber content than other cereals (18). Besides total fiber content, the composition of the dietary fiber also varies among the cereal types. Oat and barley are dominated by soluble beta-glucan, whereas the main fiber type in rye and wheat is arabinoxylan (18). Due to the overall higher content of dietary fiber, rye has approximately 50% more arabinoxylan than wheat and a larger proportion of the arabinoxylans in rye are soluble (18, 26). Soluble arabinoxylans in rye increase viscosity and are less sensitive to degradation compared with beta-glucans and may therefore exert beneficial effects on glycemic profiles and cholesterol when consumed in a wide range of products (27–29). Although observational studies have linked insoluble fiber and main sources thereof such as whole grain wheat products with reduced risk of developing type-2 diabetes and improved glycemic control, it is well established from short-term intervention studies that soluble fiber have beneficial effects on glycemic control whereas the effects on glycemia attributed to insoluble fiber are more uncertain (30, 31). An acute meal study involving 19 women showed that a meal rich in soluble fiber reduced postprandial insulin compared to a meal containing a matched amount of insoluble fiber, however, more studies directly comparing soluble and insoluble fiber in dietary intervention settings would be needed to draw strong conclusions (32).

Acute or extended meal studies investigating the effect of rye-based meals on postprandial glycemic response have repeatedly shown a pattern of reduced insulin following rye-based meals, despite similar glucose response. This phenomenon has been described and discussed as the “rye factor,” both within academia and in the industry (33), and it has been speculated whether this reduction in postprandial insulin may in part explain the mechanism behind the inverse association between rye intake and type-2 diabetes incidence (33, 34). High insulin levels have been shown to have a negative influence on the endothelial function, potentially through impaired vasodilation, which may play a role in the development of cardiovascular disease (35–37). However, not all studies investigating the postprandial response of rye-based products have supported the rye factor phenomenon and the prevalence of this phenomenon across studies and the underlying mechanisms and determinants remain unclear.

The purpose of this review was to gather studies that have investigated the acute effect of rye products on postprandial insulin and glucose and evaluate the occurrence of the rye factor phenomenon. Furthermore, we aimed to evaluate characteristics of investigated products to identify features that could help us to improve the understanding of the mechanisms behind the rye factor phenomenon. Lastly, we intended to evaluate the effect of habitual consumption of rye products on postprandial glucose and insulin response.

## Method

A search was conducted in PubMed, Scopus, and Web of Science to identify studies investigating postprandial glucose and postprandial insulin response following at least one rye product and a non-rye control product. Reference lists of known and identified articles were inspected to identify additional relevant studies. In addition to studies investigating acute postprandial effects, studies investigating effects of regular rye consumption on insulin, glucose and other known risk markers for diabetes was included. Studies on extracts or isolated fiber fractions, as well as studies including insulin dependent diabetics, were excluded. Data on identified studies were extracted into tables. If studies included several different test products, only products directly compared to the rye products(s; i.e., statistical test result reported) were included in the tables. Products with added extracts or isolated fiber fractions were not considered, as were products designed to test the effect of adding acids to a test product. Only outcomes related to glucose and insulin metabolism were considered.

Additionally, a dataset was made containing detailed information on the pairwise testing of a rye product to a non-rye product in the Postprandial phase. As several of the included studies included more than one pair of products, the same study could appear several times in this table. Similarly, a test product could appear several times, if compared to more than one other product in the original study. Since the studies evaluated the Effect on glucose and insulin in various ways, focus was put on the area under the curve (AUC) for a time period of 3–4.5 h or 2 h for studies of shorter duration and the peak values, as these measures were available for the majority of the studies. As neither AUC nor peak were reported in all studies, it was decided to use both methods in the evaluation. It was evaluated whether the rye products resulted in lower/same/higher response than the wheat product in at least one of the mentioned evaluation methods (AUC or peak). In case rye product showed higher/lower response in one the methods and no effect in the other method it was classified as lower/higher according to the method that showed effect. None of the included studies reported conflicting results (i.e., one method showing higher and the other showing lower) for any pairwise comparisons. The tested pairs were categorized as follows: “no effect” (no effect on glucose or insulin) “glucose reduction” (lower glucose, no effect on insulin), “insulin reduction” (lower insulin, no effect on glucose, i.e., rye factor phenomenon), “reducing both” (lower glucose and lower insulin), and “increase” (higher glucose and/or insulin).

## Results and Discussion

### Postprandial Effects

In total, 24 studies investigating the effects of rye products in the postprandial or extended postprandial phase were found (Supplementary Table 1). Most of the studies followed a design with a breakfast meal containing a rye product or control product served after an overnight fast. Venous or capillary blood was collected at regular intervals (every 7.5–30 min) from right before the breakfast was served and for the following 3–4.5 h. Three studies covered a shorter period of only 2 h (38–40), while two studies extended over 6 and 7.8 h and included a standardized lunch in addition to the breakfast meal (41, 42). Most of the studies consisted of more than one rye product and/or more than one non-rye product (control), and thereby consisted of more than one pairwise comparison between a rye product and a control product.

**FIGURE 1:**
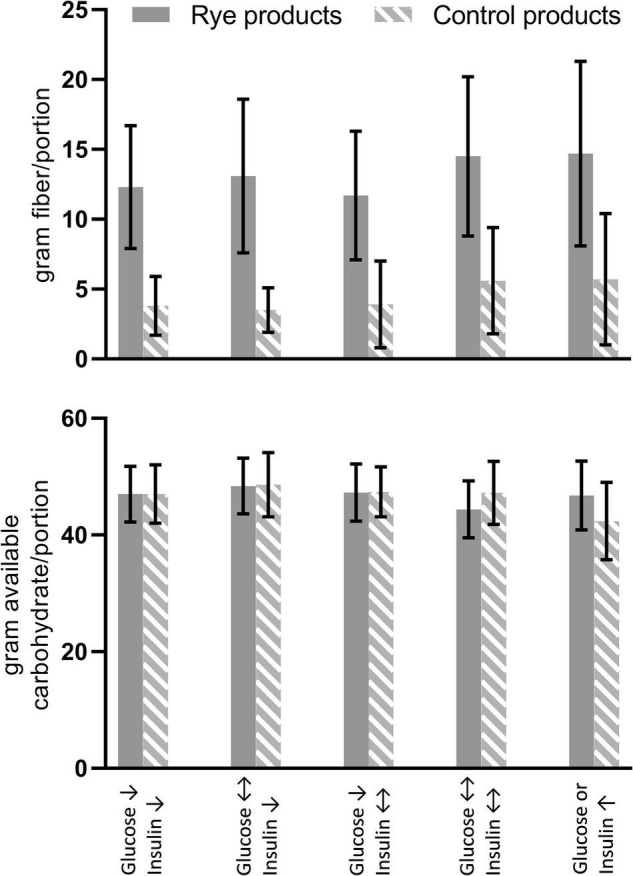
Total dietary fiber and available dietary fiber content of rye products and control products, categorized according to effect on postprandial glucose and insulin (Table 1). Data is mean and standard deviation.

From the 24 studies included in Supplementary Table 1, 72 pairwise comparisons could be extracted, and a descriptive summary of the pairs is presented in Table 1 and Figure 1. Almost all the control products were wheat based, while a few studies included barley and oat products. Most of the tested products were soft breads and the remaining could be categorized as crisp bread, cold cereal (defined as breakfast type cereals, typically eaten cold with milk, or yogurt) and porridge. The majority of the pairwise comparisons were made between the same kind of product (e.g., soft rye bread vs. soft wheat bread). However, when evaluating the processing of the cereals used in the products, most of the control products were refined endosperm-based products, whereas most of the rye products were coarser products made with whole grain rye flour or rye kernels. A large proportion of the control breads were yeast fermented breads, whereas the rye breads were a mixture of yeast and sourdough fermented breads, as well as a few unfermented breads. It should be noted that not all studies clearly report the method of fermentation of the investigated products (Table 1). Only 21 out of 72 pairwise comparisons were done between products of similar processing (e.g., endosperm wheat flour product vs. endosperm rye flour product), whereas most studies compared effects between endosperm wheat products and whole grain rye products. While rye generally has a higher dietary fiber content than other cereals (18, 43), the difference in processing has also contributed to the difference in fiber content between the rye products and the control products in the pairwise comparisons (Figure 1). The difference in available carbohydrate was low since most of the studies were standardized with regard to available carbohydrate content of the test meals. The serving size of rye products was often higher than the control product to obtain the same amount of available carbohydrate. The rye content of the rye products tested in the different studies are generally high (50–100% of cereal ingredients), except for the four bran based products, which were wheat based breads with 35% added rye bran (39, 44). Due to the higher fiber content of bran, compared to other cereal fractions, these bran-based rye breads have fiber content in similar range as the other rye-based breads (12–19 g/portion), despite the lower rye content.

**TABLE 1:** Pairs of rye-control comparisons extracted from the studies presented in Supplementary Table 1, grouped according to effect on postprandial insulin and glucose.

	Glucose ↓ Insulin ↓ [*n*: 18 (25%)]	Glucose ↔ Insulin ↓ (*“Rye factor”*) [*n*: 23 (32%)]	Glucose ↓ Insulin ↔ [*n*: 5 (7%)]	Glucose ↔ Insulin ↔ [*n*: 23 (32%)]	Glucose or Insulin↑ [*n*: 3 (4%)]
***Rye products***					
Product type	Soft bread: 16 Crisp bread: 0 Cold cereal: 0 Porridge: 2	Soft bread: 19 Crisp bread: 2 Cold cereal: 0 Porridge: 2	Soft bread: 4 Crisp bread: 0 Cold cereal: 0 Porridge: 1	Soft bread: 17 Crisp bread: 2 Cold cereal: 1 Porridge: 3	Soft bread: 2 Crisp bread: 0 Cold cereal: 0 Porridge: 1
Method of fermentation (soft bread and crisp bread only)	Yeast: 14 Sourdough: 1 Unfermented: 0 Not reported: 1	Yeast: 6 Sourdough: 9 Unfermented: 2 Not reported: 4	Yeast: 1 Sourdough: 1 Unfermented: 0 Not reported: 2	Yeast: 16 Sourdough: 2 Unfermented: 0 Not reported: 1	Yeast: 2 Sourdough: 0 Unfermented: 0 Not reported: 0
Cereal form^‡^	Bran: 0 Flakes: 0 Kernels: 7 ES flour: 3 WG flour: 8	Bran: 0 Flakes: 0 Kernels: 4 ES flour: 7 WG flour: 12	Bran: 0 Flakes: 0 Kernels: 0 ES flour: 1 WG flour: 4	Bran: 3 Flakes: 2 Kernels: 6 ES flour: 1 WG flour: 11	Bran: 1 Flakes: 1 Kernels: 1 ES flour: 0 WG flour: 0
Rye content (% w/w of cereal ingredients)^†^	81.7 ± 9.7 (75) *n* = 16	96.4 ± 10.3 (100) *n* = 17	82.0 ± 15.7 (75) *n* = 3	82.3 ± 24.5 (100) *n* = 20	67.5 ± 46.0 (67.5) *n* = 2
***Control products***					
Product type	Soft bread: 16 Crisp bread: 0 Porridge: 2	Soft bread: 19 Crisp bread: 2 Porridge: 2	Soft bread: 5 Crisp bread: 0 Porridge: 0	Soft bread: 17 Crisp bread: 2 Porridge: 4	Soft bread: 2 Crisp bread: 0 Porridge: 1
Method of fermentation (soft bread and crisp bread only)	Yeast: 16 Sourdough: 0 Unfermented: 0 Not reported: 0	Yeast: 12 Sourdough: 1 Unfermented: 0 Not reported: 8	Yeast: 1 Sourdough: 0 Unfermented: 0 Not reported: 3	Yeast: 16 Sourdough: 1 Unfermented: 0 Not reported: 2	Yeast: 0 Sourdough: 1 Unfermented: 0 Not reported: 1
Cereal form^¶^	Kernels: 2 ES flour: 14 WG flour: 2	Kernels: 1 ES flour: 20 WG flour: 2	Kernels: 0 ES flour: 4 WG flour: 1	Kernels: 5 ES flour: 14 WG flour: 4	Kernels: 1 ES flour: 2 WG flour: 0
Cereal source^¶^	16 wheat, 1 oat, 1 barley	21 wheat, 1 oat, 1 barley	5 wheat	18 wheat, 5 barley	2 wheat, 1 barley
***Rye vs. control products***					
Same product type within pair [*n* (%)]	14 (78%)	23 (100%)	4 (80%)	19 (83%)	1 (33%)
*(SB* = *soft bread* *CB* = *crisp bread* *CC* = *cold cereal* *P* = *porridge)*	*(SB-SB: 14* *SB-P_*rye*_: 2* *P-SB_*rye*_: 2)*	*(SB-SB: 19* *CB-CB: 2* *P-P: 2)*	*(SB-SB: 4* *SB-P_*rye*_: 1)*	*(SB-SB: 15* *SB-CC_*rye*_: 1* *SB-P_*rye*_: 1* *CB-CB: 2* *P-SB_*rye*_: 2* *P-P: 2)*	*(SB-SB: 1* *SB-P_*rye*_: 1* *P-SB_*rye*_: 1)*
Same cereal form within pair [*n* (%)]	6 (33%)	8 (35%)	2 (40%)	4 (17%)	1 (33%)
*(B* = *bran* *F* = *flakes* *K* = *kernels* *EF* = *endosperm flour* *WF* = *whole grain flour)*	*(K-K: 2* *EF-K_*rye*_: 4* *EF-EF: 3* *EF-WF_*rye*_: 7* *WF-K_*rye*_: 1* *WF-WF: 1)*	*(K-K: 1* *EF-K_*rye*_: 3* *EF-EF: 6* *EF-WF_*rye*_: 11* *WF-RF_*rye*_: 2* *WF-WF: 1)*	*(RF-RF: 1* *RF-WF_*rye*_: 3* *WF-WF: 1)*	*(K-K: 3* *K-EF_*rye*_: 1* *K-WF_*rye*_: 1* *EF- B_*rye*_: 3* *EF- F_*rye*_: 2* *EF-WF_*rye*_: 3* *WF-K_*rye*_: 3* *WF-WF: 1)*	*(K-K: 1* *EF-B_*rye*_: 1* *EF-F_*rye*_: 1)*
Same method of fermentation^¥^	12 of 13 (92%)	5 of 13 (39%)	1 of 1 (100%)	13 of 16 (81%)	0 of 1 (0%)
*(Y* = *yeast* *SD* = *sourdough* *UF* = *unfermented)*	*(Y-Y: 12* *Y-SD_*rye*_: 1)*	*(Y-Y: 5* *Y-SD_*rye*_: 5* *SD-Y_*rye*_-: 1* *Y-UF_*rye*_: 2)*	*(Y-Y: 1)*	*(Y-Y: 13* *Y-SD_*rye*_: 2* *SD-Y_rye_: 1)*	*(SD-Y_rye_: 1)*
*Population* (*n*: healthy/MetS/NIDD)	16/1/1	16/6/1	3/0/2	22/3/1	3/0/0

*^‡^In case a bread was composed of a mixture of rye ingredients (e.g., a rye bread with whole grain rye flour rye and rye bran) the product was categorized according to the main ingredient (weight basis). ^†^Rye content was not reported by all studies therefore n is as stated by the data and data is mean ± SD (median). ^¶^According to the main cereal ingredient (w/w%). ^¥^Only bread-bread (soft bread or crisp bread) comparisons where fermentation method was reported for both products are included. Abbreviations: MetS, metabolic syndrome; NIDD, non-insulin dependent diabetics; WG, whole grain; and ES, endosperm.*

#### The Effect of Dietary Fiber on Postprandial Responses

Generally, it should be noted that the majority of the pairs (64%) fall into categories showing a positive effect of rye in the postprandial phase (lower glucose and/or insulin response), whereas 32% show no effect and only 4% show a negative effect in the form of higher insulin or glucose following consumption of a rye product, compared to a control product (Table 1). Interestingly, the bran-based rye products fall in the latter categories showing no or negative effect of rye. Lappi et al. found a rye factor effect when comparing a whole grain rye bread to a refined wheat bread, but also when comparing the whole grain rye bread to two different rye bran breads, indicating no beneficial effect of bran based rye breads on postprandial glucose and insulin despite a dietary fiber content similar to that of the whole grain rye bread (44). This suggests that the positive effect of rye cannot be purely attributed to the high fiber content of rye products. Furthermore, most of the endosperm rye products, which have a lower dietary fiber content than other types of rye products, fall into categories supporting a positive effect of rye, further highlighting that the fiber content might not alone explain the effect. However, it should be noted that endosperm rye flour still has a higher fiber content than endosperm wheat flour, and a beneficial role of a higher dietary fiber content cannot be ruled out.

#### The Effect of Processing and Fermentation on Postprandial Responses

Although a relatively wide range of products were found in each category, we found some indications of systematic differences in the product processing and fermentation across the different categories (Table 1). The rye factor phenomenon effect was seemingly more consistent when the rye food type or processing was compared with a similar control food or processing, whereas the other effect categories had more comparisons of products of different type or processing. This could indicate that using products that differ in several factors, e.g., different processing in addition to different cereal sources, might make it difficult to disentangle the effect of rye *per se*, due to confounding by differences in structural properties or through difference in volume (45). Additionally, there seems to be a difference between the categories when looking at the fermentation method used to produce the tested breads. The rye factor phenomenon is most evident among comparisons between a sourdough fermented rye bread and a yeast fermented control product indicating that sourdough fermentation might be partly responsible for the rye factor. However, some studies using unfermented rye crisp breads (46, 47), as well studies investigating rye based porridges (39, 42), also found evidence of the rye factor phenomenon indicating that the rye factor is not restricted to sourdough fermented rye breads. Most of the pairs falling into categories of reduction in both insulin and glucose, as well as no effect on either, consist of a yeast fermented rye bread compared to a yeast fermented control bread.

#### Second Meal Effects

The second meal effect is a phenomenon where the effect of one meal extends into the following meal, e.g., an evening meal affecting our response to the following breakfast meal (48). The second meal effect is typically investigated by giving participants a test breakfast meal followed by a standardized lunch meal (morning design) or a test meal in the evening followed by a standardized breakfast meal (evening design) and monitoring the postprandial response to the standardized meal (48). A few studies have investigated the second meal effect of rye-based foods on glucose and insulin responses. Three studies using an evening design to test the second meal effect of rye based vs. wheat based control products, were identified (Table 2). The participants in these studies consumed an evening meal containing a rye product or a wheat product and the next morning the postprandial response of a standardized breakfast meal was evaluated. Additionally, two of the studies included in Supplementary Table 1 also included an evaluation of the second meal effect (41, 42). In these studies, participants consumed a breakfast meal containing a rye product or a wheat based control product, followed by a standardized lunch 4 h later, with glucose and insulin responses being monitored throughout the day (41, 42). The two studies using the breakfast design found no difference in postprandial insulin or glucose following the standardized lunch, whereas two (49, 50) of the three studies (49–51) using an evening design found lower postprandial insulin and glucose from the breakfast the day after consumption of the rye-based evening meal, compared to the wheat based evening meals. One of the studies using an evening design investigated the effect of having participants consume the rye or wheat products for three consecutive evenings before the breakfast test meal, as opposed to only consuming the rye or wheat products on the evening before the standardized breakfast test (49). However, this did not affect the results and the breakfast meal induced similar reduction in glucose and insulin response following the rye based evening meal, compared to the wheat based evening meal, independent on whether the evening meals had been consumed one or three evenings leading up to the breakfast meal (49).

**TABLE 2:** Second meal effect studies.

	Design*	Subjects^‡^	Intervention products*	Study procedure	Outcomes (data analysis)*	Results Mean ± SEM, unless otherwise stated
Sandberg et al. (50)	Cross-over, randomized, two test meals	38 (30 f/8 m), age 63.9 ± 5.5 year, BMI 24.2 ± 2.5 kg/m^2^, fasting glucose 5.7 ± 0.4 mmol/l, no known metabolic disease, non-smoking.	Rye bread with added resistant starch (RBRS):43% rye kernels, 43% whole grain rye flour 14% Hi-Maze flour (60% resistant starch type 2, 40% digestible starch). White wheat bread (WWB): 100% refined wheat flour. Composition rye bread/wheat bread (g/portion): portion size 239/171, starch 89/76 (available starch 75/75), insoluble DF 21/4, soluble DF 6/1.	Subjects consumed one portion of test bread per day for three days before visiting the clinic. On day 1 and 2 bread was divided over the day, on day 3 the portion was consumed at 9 pm. At the clinic subjects consumed 114 g WWB 50 g available starch with 2 dl water after an overnight fast (from 9 pm). Blood was taken at 0, 15, 30, 45, 60, 90, 120, and 180 min.	Glucose (capillary blood), insulin (venous blood). iAUC_0–30_, iAUC_0–120_, incremental peak, Composite Insulin Sensitivity Index (ISI_*composite*_), HOMA-IR.	Glucose: iAUC_0–30_ lower following RBRS, compared to WWB (−14%, *p* < 0.05). Insulin: Incremental peak lower following RBRS, compared to WWB (−15%, *p* < 0.01). ISI_*composite*_ higher following RBRS, compared to WWB (+11%, *p* < 0.05).
Sandberg et al. (51)	Cross-over, randomized, three test meals	21 (10 m/11 f), age 25.3 ± 3.9 year, BMI 22.7 ± 2.3 kg/m^2^, no known metabolic disease, non-smoking.	Whole grain rye flour bread (RFB): 100% whole grain rye flour. Whole grain rye flour and rye kernel bread (RKB): 50% whole grain rye flour, 50% rye kernels White wheat bread (WWB; reference): 100% refined wheat flour. Composition of RFB/RKB/WWB (g/portion): portion size 187/184/122, starch 53/57/52 (available starch 50/50/50, RS 3/7/1), insoluble NSP 11/13/2, soluble NSP 4/4/1, total DF 18/23/4.	Subjects consumed test breads at 9 pm the night before visiting the clinic. At the clinic subjects consumed one portion WWB with 2 dl water after an overnight fast (from 9 pm). Capillary blood taken at 0, 15, 30, 60, 90, 120, 150, and 180.	Glucose (all timepoints), insulin (all timepoints except 15 min) iAUC_0–120_, incremental peak.	No difference
Sandberg et al. (49)	Cross-over, randomized, four test meals (2 × 2 factorial design)	19 (9 m/10 f), age 21.9 ± 1.87 year, BMI 25.6 ± 3.5 kg/m^2^, no known metabolic disorders, non-smoking.	White wheat bread (WWB; reference): 85% rye kernels and 15% white wheat flour (of cereal dry matter). 121.4 g bread/portion. Rye kernel bread (RKB): 100% white wheat flour. Composition WWB/RKB [g/portion (142.5 g)]: starch 51.3/53.9 (available starch 50/50, resistant starch 1.3/4.9), insoluble NSP 1.5/9.0, soluble NSP 1.1/2.8, DF 3.9/15.6.	Subjects consumed one portion of test bread with water at 9:30 pm either: the day before visiting the clinic (WWB-1D or RKB-1D) of for three consecutive days before visiting the clinic (WWB-3D or RKB-3D). At the clinical visit the subjects consumed 121.4 g WWB with 2 dl water after an overnight fast (from 9:30 pm). Venous blood drawn at 0, 15, 30, 45, 60, 90, 120, and 180 min.	Glucose (all timepoints), insulin (all timepoints except 15 min) iAUC_0–120_, incremental peak, Matsuda index (insulin sensitivity).	No effect of length of priming (1 or 3 days) were found in any of the outcomes.Glucose: iAUC_0–120_ and incremental peak was lower following a RKB evening meal than a WWB evening meal (23 and 16%, respectively, *p* < 0.01). Insulin: iAUC_0–120_ was lower following a RKB evening meal than a WWB evening meal (13%, *p* < 0.05)

^‡^Data is mean ± sd or (range). ^*^Only test meals and outcomes of interest for investigating the rye factor (insulin, glucose) is included in the table. Abbreviations: BMI, body mass index; DF, dietary fiber; f, female; HOMA-IR, homeostatic model assessment for insulin resistance; iAUC, incremental area under the curve; m, male; and NSP, non-starch polysaccharides.

These studies indicate that an evening meal containing rye may have a beneficial effect on glycemic control at the following breakfast meal, but none of these studies showed a postprandial response in line with the rye factor phenomenon.

### Effects of Habitual Rye Consumption on Glycemic Control

While many studies investigating the acute postprandial effect of rye consumption have been conducted and in general show positive effects on postprandial glucose and insulin responses, only a few randomized intervention studies have investigated the effect of habitual rye consumption on postprandial glycemic control. Five studies investigating the effect of habitual rye consumption on markers of glycemic control were identified and are shown in Table 3. These studies consist of four randomized cross-over studies with a duration of 4–8 weeks (27, 52–54), as well as a 12-week randomized parallel study (55). The studies all included an intervention arm based on rye products, whereas the products included in the control arm(s) vary between the studies, but typically contained wheat products. The result from these studies show mixed results, with some studies showing a positive effect of rye consumption (52, 55), whereas others show no or negative effect (27, 54). Lappi et al. found a positive effect of whole grain rye bread on postprandial insulin response after 4 weeks of intervention, compared to 4 weeks intervention with refined wheat bread, whereas no such effect was found after 4 weeks of intervention with refined wheat bread with added rye bran (52). No difference in glucose response was found between either of the arms, indicating a pattern in agreement with the rye factor phenomenon. The lack of a positive effect of the rye bran-based intervention arm is in line with observations from postprandial studies, where similar types of rye bran bread did not lower postprandial insulin or glucose response and could indicate that the fiber content is not the sole explanation of the positive effects of rye consumption. However, McIntosh et al compared high fiber rye products to low fiber wheat products, as well as to high fiber wheat products, in a three armed 4-week intervention study, and found a positive effect of both high fiber rye and high fiber wheat, compared to refined wheat, but no difference between the two high fiber arms (53). On a similar note, Eriksen et al. found no difference in an oral glucose tolerance test after 4 and 8 weeks of consumption of whole grain rye products or whole grain wheat products matched for fiber content, indicating no difference between the sources of cereals under conditions where the fiber content of the two intervention diets were matched (27). On the other hand, Laaksonen et al. found reductions in insulinogenic index and insulin disposition index in an oral glucose tolerance test following a 12-week intervention with rye products and pasta, compared to 12-week intervention with oat, wheat and potato based products with a similar fiber content (55). However, there were no differences in fasting insulin and glucose, as well as AUC_0–120 *min*_ of glucose and insulin in the oral glucose tolerance test (55). Furthermore, it should be noted that the products included in the intervention arms included pasta and potatoes in addition to several different breads, making it hard to pin the results exclusively to the rye products (55).

**TABLE 3:** Studies investigating the effect on habitual consumption of rye products on glycemic control.

	Design*	Subjects^‡^	Intervention products*	Study procedure/clinical examinations*	Outcomes (data analysis)*	Results Mean ± SEM, unless otherwise stated
Eriksen et al. (27)	Cross-over, randomized, two intervention period (8 + 8 weeks, separated by 8-week wash-out).	49 men, age 49–74, BMI 26–41 kg/m^2^, signs of metabolic syndrome.	Rye period: breakfast cereals, crisp bread and pasta based on whole grain rye. Wheat period: breakfast cereals, crisp bread and pasta based on whole grain wheat with added wheat bran to match the fiber content of rye products. Products were aimed to constitute 30% of daily energy intake.	OGTT (75 g) in the beginning, middle and end of each intervention period (week 0, 4, and 8). Intravenous blood was drawn at 0, 30, 60, and 120 min.	Glucose, insulin. AUC_0–120_ glucose and insulin.	No effect.
Lappi et al. (52)	Cross-over, randomized, 4 weeks run-in period and two intervention periods (4 + 4 weeks). No wash out period.	21 males and females, age 38–65 year, BMI 19–30 kg/m^2^, fasting glucose 4.9–6.3 mmol/l.	Refined wheat bread (WW; run-in period): two commercial breads with 100% white wheat flour, 20–35 g/slice. Whole grain rye (WGR; intervention period): sourdough fermented whole grain rye bread, 25–30 g/slice. Wheat bread with bioprocessed rye bran (WWBRB; intervention period): white wheat bread with 35% (dry matter) bioprocessed rye bran, 25–30 g/slice. Subjects were instructed to consume 6–10 slices bread/day. Reported intake per day of WW/WWBRB/WGR: 169/195/205 g bread, 5/20/21 g DF from bread.	3-h meal test at the end of run-in period and end of each intervention period. Meal (80 g WW bread, 20 g cheese, 40 g cucumber, 3 dl juice. 550 kcal, 3.7 g DF) was consumed after an overnight fast. Venous blood samples were collected at 0, 30, 60, 120, and 180 min.	Glucose, insulin Fasting values, iAUC_0–120_, first-phase insulin secretion (0–30 min), insulin disposition index.	Glucose: No difference Insulin: WGR lower at 120 min, compared to WW (*p* = 0.023). Disposition index higher after WGR, compared to WW (3,614 ± 2,883 vs. 2,500 ± 1,336, *p* = 0.033).
Laaksonen et al. (55)	Parallel, 4-week run-in (habitual diet), hereafter randomized to one of two 12-week intervention arms.	72 (36 m/36 f), metabolic syndrome, age 40–70 year, BMI 26–40 kg/m^2^. 65% had impaired fasting glucose, 42% had impaired glucose tolerance.	Oat-wheat-potato (OWP) group: wheat bran bread, graham crisp, graham toast and oat bread (60% whole meal oat). Rye-pasta (RP) group: two whole meal rye breads, whole meal rye crisp bread, endosperm rye bread. Subjects were instructed to replace habitual bread with test breads. Furthermore, subjects in RP was instructed to consume ≥3 portions (min 210 g/week) dark pasta per week. Subjects in OWP were instructed to eat similar amount of potatoes. Reported intake OWP/RP: 247/244 g bread/day, potato products 4.4/2.9 times/week, pasta 0.7/2.9 times/week, energy 7.9/8.3 MJ/day, DF 21/26 g/day.	OGTT at baseline and after 12 weeks. Glucose solution (75 g glucose) consumed after an overnight fast. Blood drawn at 0, 15, 30, 45, 60, 90, and 120 min.	Glucose, insulin Fasting values, QUICKI, insulinogenic index (IGI), insulin disposition index (DI), AUC	IGI and DI increased more in the RP group, than in the OWP group (approx. 30% vs. 5%, *p* = 0.026–0.030)
McIntosh et al. (53)	Cross-over, randomized, three intervention periods (4 + 4 + 4 weeks).	28 males, age 40–65 year, no gastrointestinal disorders, BMI 30 ± 0.9 kg/m^2^.	Intervention product per day (for 4 weeks): Low fiber diet: 140 g refined wheat bread, 40 g refined wheat crisp bread, 50 g low fiber rice cereal (19 g DF/day). High fiber wheat diet: 140 g whole meal bread, 40 g whole meal wheat crisp bread, 50 g whole wheat breakfast cereal (32 g DF/day). High fiber rye diet: 140 g whole grain rye bread, 40 g rye crisp bread, 50 whole-rye breakfast cereal (32 g DF/day).	1-h meal tolerance test at the end of each 4-week period. Subjects consumed one portion of breakfast cereal according to randomization (50 g available CHO) with 1 dl milk after an overnight fast. Venous blood drawn at 0 and 60 min.	Glucose, insulin. Fasting and postprandial. Fasting values, Δ_0–60 *min*_	Glucose: Δ_0–60 *min*_ lower after high fiber diets, compared to low fiber diet (1.35 ± 0.3, 0.95 ± 0.2, 2.42 ± 0.4, *p* < 0.0005)*. Insulin: Δ_0–60 *min*_ lower after high fiber diets, compared to low fiber diet (19.6 ± 2.1, 20.8 ± 2.8, 48.9 ± 6.5, *p* < 0.0001)*. *high fiber rye, high fiber wheat, low fiber.
Juntunen et al. (54)	Cross-over, randomized, two intervention periods (8 + 8 weeks). Intervention was preceded by 2-3-week run-in period and separated by an 8-week wash-out period (both habitual diet).	20 postmenopausal women, healthy, age 59 ± 6.0 year, BMI 27.5 ± 2.9 kg/m^2^.	Subjects were instructed to replace habitual bread with intervention breads during the 8-week intervention periods. Subjects were instructed to consume at least 4–5 portions of bread per day (20–28 g/174–249 kcal per portion). Rye period: subjects could choose from four different rye breads with similar nutrient composition (≈19% DF) Wheat period: Subjects could choose from seven different wheat breads produced from refined wheat flour (≈2.8% DF)	Frequently sampled intravenous glucose tolerance test (FSIGTT) was conducted at baseline and after each intervention period. Glucose dose of 330 mg/kg body was infused, and intravenous samples taken at 0, 2, 4, 6, 8, 10, 12, 14, 16, 19, 22, 24, 27, 30, 40, 50, 60, 70, 90, 100, 120, 140, 160, and 180 min.	Glucose, insulin. Fasting values. Repeated measures, glucose effectiveness and insulin sensitivity. Acute insulin response (AIR) calculated as AUC_0–10_.	The increase in AIR (compared to baseline) was higher in the rye period (9.9 ± 24.2%) than in the wheat period (2.8 ± 36.3%).

^‡^Data is mean ± sd or (range). *Only test meals and outcomes of interest for investigating the rye factor (insulin, glucose) is included in the table.

In summary, there is no consistent evidence supporting a positive effect of habitual rye consumption on postprandial glycemic control, despite the predominantly positive effect found in acute meal studies. However, it should be mentioned that most of these studies evaluate the effect of the intervention on oral glucose tolerance, which may not be directly comparable to a meal tolerance test such as the ones typically used in the acute meal studies. Furthermore, the relatively large variations in study duration, outcomes assessed, and intervention products used makes it difficult to draw firm conclusions and further studies are needed to understand the potential link between the positive effects from acute meal studies and the long-term associations with improvements in glycemic control and reduced risk of type-2 diabetes.

### Potential Mechanisms Behind the Rye Factor

Several mechanisms behind the rye factor phenomenon have been suggested and discussed (33). One suggested mechanism is related to structural differences in the cereal products resulting in slower glucose uptake in the gastrointestinal tract following consumption of a rye-based cereal product, compared to a non-rye control product (54, 56). If glucose uptake is slower, less insulin would be needed to maintain a similar concentration of glucose in the blood and therefore the insulin secretion is lower following consumption of the rye-based product, while the glucose response is similar to the control product. This hypothesis was tested by Östman et al. who studied the glucose kinetics using a dual tracer technique, as well as glucose and insulin response, to a rye bread and a wheat bread in a dual isotope labeling study (57). They found that the rye factor phenomenon was related to slower uptake of glucose in the intestine but not to altered disappearance or clearance of glucose in the blood stream.

Furthermore, high fiber cereals have been shown to increase the fecal energy excretion, through binding of nutrients from the food and reduce the absorption (58). Therefore, it could be theorized, that even though the studies match the rye and control products in terms of available carbohydrate, a larger amount of the available carbohydrate will be bound to the dietary fiber matrix in the fiber-rich rye products, compared to the control products which typically has a lower fiber content, and therefore the amount of carbohydrate available for absorption in the intestine will in reality be lower for rye foods, despite similar content. This could then lead to a slower and/or reduced intestinal absorption of glucose and subsequent reduced insulin secretion.

Different fermentation methods affect the structure of bread, which could in turn be thought to influence the metabolic response to bread consumption (59, 60). Two studies have tested the effects of unfermented rye crisp bread and sourdough or yeast fermented rye crisp bread on postprandial glucose and insulin response. They found lower postprandial insulin response following consumption of the unfermented crisp bread and the authors suggest that this could be due to structural differences in the unfermented breads, compared to the fermented breads (46, 47). A recent study showed that increasing the sourdough content in rye bread affected the structure of the bread and that bread with higher sourdough content resembled refined wheat bread more than a bread with lower sourdough content (61). One could hypothesize that this would lead to higher postprandial glucose and insulin response, but this remains to be tested *in vivo*. Additional studies investigating the effect of different fermentation methods on structural properties of bread is needed in order to fully understand the effect of fermentation on structure and potentially link it to the physiological response *in vivo*.

Branched chain amino acids (BCAA) have been shown to induce insulin secretion (62) and it has been speculated whether the amino acid composition and content of cereal products may influence the postprandial response (46). Generally, the content of amino acids, including BCAA, seem to be higher in the bran faction of the cereal, compared to whole grain flour and sifted flour (63), which could potentially contribute to the lack of a positive effect of bran based rye breads on postprandial insulin and glucose. However, the amino acid content is also highly affected by baking and fermentation, as well as cultivation, why more research is needed in order to understand the potential effect of cereal amino acids on postprandial response (63).

Rye contains several different bioactive compounds that have been suggested to have positive effects on various health outcomes, such as glycemic control and insulin sensitivity (33). Lignans and alkylresorcinols have been shown in *in vitro* and animal studies to improve insulin sensitivity (64, 65). An association between alkylresorcinols and insulin sensitivity has been confirmed in human studies (29, 66), but since alkylresorcinols may just be a marker of whole grain rye and wheat intake, it is difficult to distinguish between the effect of the alkylresorcinols and other components of whole grain products that could influence insulin sensitivity (67). Human studies on lignans and health has primarily been focused on cancer, due to the potential mild estrogenic effects of certain lignan metabolites (68). The few studies that have investigated the potential link between lignan intake and outcomes related to glycemic control and type-2 diabetes have found conflicting results (69) and as with alkylresorcinols it is difficult to distinguish between the effect of lignans and the effect of other potentially beneficial components of rye. Furthermore, it should be noted that lignans are found in relatively high amounts in other foods, such as flaxseeds, and the associations found in observational studies is not necessarily reflecting an association with lignans from rye (70).

If the mechanism behind the rye factor is mainly related to fiber content and structure, a rye factor phenomenon could likely be obtained with other cereals than rye. Eelderink et al. used a similar methodology as Östman et al. to examine the postprandial response to two wheat based breads with similar ingredients, but different structure (a standard sliced loaf bread and a flat bread; 57, 71). While glucose response was similar between the breads, insulin response was lower for the flat bread, compared to the loaf bread, and glucose kinetics revealed a slower uptake of glucose in the intestine from the flat bread, which likely explains the lower insulin response. In a similar study, comparing two different loaf type wheat breads with different structure and fiber content, Eelderink et al. found similar, but less pronounced results (72). Goletzke et al. found that a whole meal spelt bread had similar effects as two rye based breads, compared to a low fiber soft wheat pretzel (38). Liljeberg et al. found lowering of insulin, without affecting glucose, when comparing barley kernel based breads and a rye kernel based bread with a wheat kernel based bread (73). Together, these findings suggest that the rye factor phenomenon is not restricted to rye-based products. However, rye and barley have higher contents of soluble fiber than wheat, which will likely affect the structure and digestion of the cereal products and may explain the similarities in the physiological response. Bran has more insoluble fiber, and less soluble fiber, than endosperm and whole grain flour which may explain the lack of a beneficial effect of bran-based rye breads on postprandial insulin and glucose (39, 44).

In summary, limited evidence for the underlying mechanism behind the rye factor exist, but it appears that structural properties, such as particle size, viscosity, and fiber matrix, are likely the major determinants for the postprandial response to consumption of cereal products.

### Limitations and Future Perspectives

Amongst the studies included in this review there is a large variation in the methodology used to evaluate the postprandial response to the tested products. Some studies report the results of repeated measures models, some report AUC or iAUC over time periods varying between 2 and 5 h and some used other measures, such as peak values, glycemic index and insulin disposition index. This makes it impossible to directly compare the effect size between different studies and give an overall estimate on the magnitude of the effect across studies. While postprandial glucose response has been shown to correlate with glycated hemoglobin, which is a stronger predictor of diabetes risk, little is known about the long term implications of postprandial insulin response (74). Some studies have indicated an association between postprandial insulin response and risk of metabolic disease (75, 76), however, more evidence is needed in order to evaluate the implications of reductions in postprandial insulin response on long term disease risk.

Our understanding of the mechanism of action behind the effect of different cereals on postprandial glucose and insulin response remains suggestive, as only few studies include more mechanistic outcome measures such as measures of glucose kinetics using tracer techniques. Studies by Eelderink et al. and Östman et al. shed some light on a potential underlying mechanism by incorporating tracer-techniques, but more studies are needed to draw general conclusions (57, 71, 72).

The degree of characterization and the reporting of compositional information on products vary between studies and makes it hard to make comparisons across studies. Generally, more detailed characterization beyond nutritional values, is needed to further elucidate the effects of rye products on glycemic control as well as the underlying mechanisms. Furthermore, many studies compare rye products with a relatively high fiber content to wheat-based products with a relatively low fiber content, which makes it difficult to distinguish between the effect of fiber content and other factors, such as cereal source. However, it is important to keep in mind that the different cereals have a different fiber content and composition, even in their native form (18), why there will be differences in the fiber content, even when comparing a 100% whole grain rye product to a 100% whole grain wheat product – which underlines that fiber content and composition may explain part of the rye factor phenomenon. Moreover, one should consider the effect of different processing techniques, e.g., a milled whole grain rye flour compared with rolled whole grain rye flakes which may have very similar fiber content and composition, but at the same time express vastly different structural properties, which may in turn affect the physiological response (77).

Method of fermentation has been shown to influence the structure of bread, which could in turn influence the physiological response to consumption of the breads. However, this is often clouded by the fact that sourdough fermented rye breads are often compared to yeast fermented wheat breads, making it difficult to disentangle the effect of cereal source from the method of fermentation. From an applicational point of view it is sensible to test a yeast fermented wheat bread with a sourdough fermented rye bread, as this is the typical types of bread available on the market and consumed by consumers. However, from a mechanistic point of view studies investigating different combinations of cereal sources and methods of fermentation are needed in order to understand the underlying mechanisms and determinates for postprandial response.

## Conclusion

In conclusion, rye-based products have consistently been shown to lower insulin response in the postprandial phase, either alone or in combination with reductions in glucose response, compared to wheat-based products. Recent mechanistic studies using tracer-techniques have suggested that this may be attributed to slower glucose uptake in the intestine, which in turn may be linked to structural properties of rye products, rather than fiber content *per se*. However, the rye factor phenomenon might not be a unique property of rye-based cereal products, as similar effects have been observed from other types of cereal products with similar fiber content and structural properties. Some studies indicate a role of sourdough fermentation, potentially through its effect on structure, but due to lack of studies properly designed to address this research question, effects of fermentation from other factors cannot be disentangled. There is a need for carefully controlled studies investigating the postprandial effects of products based on different cereals, as well as the structural properties standardized across cereal species to understand the link between structural properties and postprandial response.

## Author Contributions

RL conceived the idea for the review. KI and KJ conducted the literature search and extracted the data. KI analyzed the data and wrote the article under supervision of RL. All authors read the manuscript, provided valuable inputs, and approved the final manuscript.

## Conflict of Interest

RL is the founder of the Nordic Rye Forum, which is a research and dissemination platform for research related to rye and health that includes academic institutions as well as institutes and food industry with interest in rye across the Nordic region. The forum and its activities are funded by the industrial partners. RL is the PI of several projects funded by several cereal industrial companies. Such funding is used to carry out scientific studies. RL receives no salary, honorary, or by any other means have any personal economic benefits from industrial collaborations. The remaining authors declare that the research was conducted in the absence of any commercial or financial relationships that could be construed as a potential conflict of interest.

## Publisher’s Note

All claims expressed in this article are solely those of the authors and do not necessarily represent those of their affiliated organizations, or those of the publisher, the editors and the reviewers. Any product that may be evaluated in this article, or claim that may be made by its manufacturer, is not guaranteed or endorsed by the publisher.
