# Supplementary material for: The Effect of Rye-Based Foods on Postprandial Plasma Insulin Concentration: The Rye Factor
Source: Front Nutr. 2022 Jun 9;9:868938. doi: 10.3389/fnut.2022.868938 (PMC9218669; doi:10.3389/fnut.2022.868938)
Supplement: Supplementary file 1 [file Table_1.docx]

**Table 1:** Acute, postprandial studies (breakfast design). All studies were randomized unless otherwise stated

|  | **Design, number of test meals*** | **Subjects^ǂ^** | **Intervention products*** | **Study procedure** | **Outcomes (data analysis approach)*** | **Results** |
| --- | --- | --- | --- | --- | --- | --- |
| Östman et al 2019 (57) | Cross-over, 2 test meals | 9 m, nonsmoking, age 18-40 y, BMI 22.1-25.2 kg/m^2^, normal blood pressure, normal fasting glucose, normal blood lipids. | Rye bread: Traditional whole meal rye bread consisting of whole meal rye flour and rye sourdough.  Wheat bread: commercial yeast fermented wheat bread, with endosperm wheat flour.  Composition wheat bread/rye bread (per serving): portion size 121/148g, available CHO 50/50g, starch 69/57 % of DM, soluble DF 1/4 % of DM, insoluble DF 3/12 % of DM | Bread was served with 50g cucumber and 2.5 dl water after an overnight fast.  Venous blood was drawn at 0, 15, 30, 45, 60, 75, 90, 120, 150 and 180 min. | Glucose, insulin  Repeated measures, AUC_0-90_ | Glucose:  No difference  Insulin:  Rye was lower at 60 and 75 min, compared to wheat. AUC_0-90_ was lower for rye than for wheat. |
| Lee et al 2017 (41) | Cross-over, 3 test meals | 21 (11 m/10 f), healthy, age 38.6±11.8 y, BMI 24.9±3.3 (range 21-33) kg/m^2^. | Wheat bread (WB) (reference): 55g refined wheat bread, 12 g margarine.  Rye porridge (RP40): 40g rye whole grain rye flakes, 17g margarine, 150g water.  Rye porridge (RP55): 55g whole grain rye flakes, 4g margarine, 200g water.  Meals also included 25g jam, 100g milk and 150g tea/coffee.  Meal composition per portion (WB/RP40/RP55): energy 1158/1191/1205 kJ, fat 8/9/4g, protein 10/7/8g, CHO 39/41/50g, DF 3/7/10g. | Meal served after an overnight fast. Standardized lunch (sausage stew with rice) served after 240 minutes.  Venous blood drawn at -15, 15, 35, 65, 95, 125, 185, 230, 275, 305, 365 and 470 min. | Glucose, insulin  AUC_-15-125_, AUC_-15-230_, AUC_275-470_, AUC_-15-470_. | Glucose:  RP55 higher AUC_-15-125_ than RP40 and lower AUC_275-470_ than WB.  Insulin:  No difference |
| Zamaratskaia et al 2017 (47) | Cross-over, 3 test meals | 24 (13 m/11 f), healthy, mean age 30 y, mean BMI 23 kg/m^2^, fasting glucose <6.0 mmol/l. | WCB: Yeast fermented wheat crisp bread (refined wheat flour).  uRCB: Unfermented rye crisp bread (whole grain rye flour).  sRCB: Sourdough fermented rye crisp bread (whole grain rye flour).  Test meals also included margarine, cheese and orange juice.  Nutrient composition of test meals WCB/uRCB/sRCB (g/portion)  Amount 187/195/195; fat 15/13/13; protein 12/12/11; CHO 44/45/46; DF 4/12/10; energy 1528/1523/1514. | Test meals served after an overnight fast.  Venous blood sample drawn at 0, 15, 35, 50, 65, 95, 125, 155, 185 and 230 min. | Glucose, insulin  Repeated measures. AUC_0-125_ and AUC_0-230_. | Glucose:  No difference.  Insulin:  AUC_0-125_: uRBC was lower than sRBC and WCB. |
| Nordlund et al 2016 (78) | Cross-over.  **Series 1:** 2 test meals.  **Series 2:** 4 test meals.  **Series 3:** 3 test meals.  B1 used as reference in all series. | Series 1: 17 men and women, age 22–37 y, BMI 19.2–26.7 kg/m^2^; normal glucose tolerance.  Series 2 and 3: 12 females, age 64 y (range 57–72), body weight 68.3±8.4 kg, normal glucose tolerance | B1 (reference): commercial refined wheat bread.  B2: commercial whole meal sour rye bread with finely milled flour.  B3: 100% whole meal rye flour bread, with sourdough.  B4: 70% whole meal rye flour, 30% rye bran, with sourdough.  B5: 100% refined rye flour, with sourdough  B6: Same as B5 but baked flat.  Composition of breads B1/B2/B3/B4/B5/B6 (g/100g)  Portion size (g) 99/135/143/199/112/91; sugars 2/5/0.3/0.2/0.4/1; available CHO 50/37/35/25/45/55; DF 2/9/11/15/6/6; (hereof soluble DF 0.5/1.6/3/2.4/2.1/2.4); protein 9/6/8/8/4/4; fat 5/1/6/5/0.7/0.4. | Breads (50g available CHO/portion) served with 40g cucumber and 3dl non-caloric beverage after an overnight fast.  Blood samples drawn at 0, 15, 30, 45, 60, 90, 120, 150 and 180 min. | Glucose,  Insulin.  iAUC | **Series 1 (B1 and B2):**  Glucose: No difference.  Insulin: B2 lower than B1.  **Series 2 (B1, B3, B4, B5):**  Glucose: No difference  Insulin: B3 and B5 lower than B1  **Series 3 (B1, B6):**  Glucose: No difference  Insulin: B6 lower than B1 |
| Goletzke et al 2016 (38) | Cross-over, 4 test meals | 12 (5 f/7 m), Caucasian, age 26.3±5.7 y, BMI 22.3±2.6 kg/m^2^, fasting glucose 5.22±0.05 mmol/l, recruited within panel of regular participants at the Sydney University Glycaemic Index Research Service. | Whole meal rye bread with intact kernels and sunflower seeds (RK).  Rye wheat sourdough bread (RWS).  Soft pretzel (SP) (reference).  Test portions of RK/RWS/SP (g):  152/119/100 (50g available CHO).  Composition (/100g) of RK/RWS/SP: energy 248/216/279 kcal, CHO 33/42/50g, DF 8/6/na g, fat 8/1/5g, protein 8/7/6/8g. | Conducted in accordance with ISO standard protocol for GI determination.  Breads were served with 250 ml of water after an overnight fast.  Blood were drawn at 0, 15, 30, 45, 60, 90 and 120 min. | Glucose, insulin  iAUC, GI, II | Glucose:  SP had higher iAUC and GI than RK and RWS.  Insulin:  SP had higher II than RK and RWS. RWS did not differ from other breads. |
| Johansson et al 2015 (46) | Cross-over, 3 test meals | 23 (7 m/ 16 f), age 60±12 y, BMI 24±10 kg/m^2^, healthy, fasting glucose <6.0 mmol/l. | WCB: Yeast fermented wheat crisp bread (refined wheat flour).  uRCB: Unfermented rye crisp bread (whole grain rye flour).  yRCB: Yeast fermented rye crisp bread (whole grain rye flour).  Test meals also included margarine, cheese and orange juice.  Composition of test breakfasts for uRCB/yRCB/WCB (g/portion)  Amount 194/195/187; fat 13/13/16; protein 11/11/12; CHO 46/47/44; DF 12/11/4; energy (kJ) 1538/1561/1561. | Test meals served after an overnight fast.  Venous blood sample drawn at 0, 15, 35, 65, 95, 125, 155, 185 and 230 min. | Glucose, insulin  Repeated measures.  AUC_0-125_, AUC_0-230_ | Glucose:  No difference  Insulin:  WCB higher than yRCB and uRCB at 65 and 95 min.  uRCB had lower UAC_0-230_ than yRCB and WCB.  In AUC_0-120_ all three crisp breads differed from one another (WCB>yRCB>uRCB). |
| Hartvigsen, Lærke et al 2014 (42) | Cross-over,  2 test meals. | 15 (8 m/7 f),  metabolic syndrome, age 63.5±5.0 y (range 52-73); weight 95.0±12.1 (80.4-111.9) kg; BMI 31.3±2.7 (26.2-34.7) kg/m^2.^ | SE (reference): semolina porridge (69g semolina, 1g salt, 2 pinch stevia)  RK: rye kernel porridge (28g semolina, 50g rye kernels, 1g salt, 2 pinch stevia)  Per portion (SE/RK): 525/435g, 1105/1001 kJ, 50/50g digestible CHO, 8.1/7.3g protein, 0.7/1.1g fat, 5.7/12.7g fiber, 1.8/1.4g resistant starch, 3.3/9.1g NSP. | Test meals (SE or RK) served following an overnight fast.  Blood samples drawn at 0, 15, 30, 45, 60, 90, 120, 240, 255, 270, 285, 300, 330, 360 min.  Standardized lunch (refined wheat bread) served after 240 min (second meal effect). | Glucose, insulin.  Time x treatment interaction for whole day.  iAUC_0-120_ and iAUC_240-360_. | Glucose:  No difference  Insulin:  RK lower iAUC_0-120_ than SE. |
| Hartvigsen, Gregersen et al 2014 (79) | Cross-over, 2 test meals | 15 Caucasian (7 m/8 postmenopausal f), metabolic syndrome, age 52-72 y, BMI 31.1±3.2 kg/m^2^. | Commercial rye bread: whole grain rye kernels (49% of dough), water, rye sourdough, rye breads crumbs.  Commercial wheat bread: refined wheat flour (68% of dough), yeast fermented.  Composition wheat bread/rye bread (g/serving): portion size 107/147, protein 9/7, fat 2/2, available CHO 50/50, DF3/12 (NSP 2/11), energy 1088/1040 kJ. | Bread was served with 3 dl water after an overnight fast.  Venous blood drawn at 0, 15, 30, 45, 60, 90, 120, 180, 240 and 270 min. | Glucose, insulin  iAUC_0-120_, iAUC_0-270_, GI, II, repeated measures | Glucose:  iAUC_0-120_  and GI lower for rye bread, compared to wheat bread.  Rye lower than wheat at 45, 60 and 90 min.  Insulin:  iAUC_0-120_, AUC_0-270_ and II lower for rye bread, compared to wheat bread. Rye bread lower than wheat bread at 45, 60, 90 and 120 min. |
| Breen et al 2013 (80) | Cross-over, 4 test meals | 10 (6 men, 4 f), type-2 diabetics, age 53.9±5.5 y, BMI 35.1±7.5 kg/m^2^, HbA1C 6.3±0.4%. None of the subjects received pharmaceutical treatment for diabetes, but majority were using lipid lowering and/or blood pressure lowering medication. | All breads were commercially available.  Wheaten white (WW): White wheat, yeast fermented.  Stone-ground whole wheat butter milk bread (WB): Whole wheat flour, buttermilk.  Wheaten whole grain (WG): whole meal wheat flour and cracked wheat.  Pumpernickel (PB): rye-based pumpernickel bread.  Composition WW/WB/WG/PB (g/portion): portion size 124/131/140/196, total CHO 53/58/58/69 (available CHO 50/50/50/50), protein 11/10/13/10, fat 2/2/3/4, DF 3/7/8/19, energy 1145/1233/1364/1492 kJ. | Bread was served with 2.5 dl water after an overnight fast.  Capillary (glucose) and venous (insulin) blood drawn at -15, 0, 15, 30, 45, 60, 90, 120, 150, 180, 210, 240 and 270 min. | Glucose, insulin  iAUC, repeated measures, peak. | Glucose:  PB had lower iAUC than WG.  PB had lower peak than WW, WB and WG.  Insulin:  iAUC was lower for PB than for WW and WG but did not differ from WB.  Peak concentration was lower for PB than for WW and WG. |
| Lappi et al 2013 (44) | Cross-over, 4 test meals | 15 (9 f/6 m), age 57 y (35-65 range), BMI 26 (21-32) kg/m^2^. Mild/moderate/severe self-reported gastrointestinal symptoms from cereals, particularly rye bread (used for aims in other study). | White wheat bread (WW) (reference). Commercial rye bread with 100% whole grain rye flour (CR).  White wheat bread fortified with native rye bran (RB+WW). White wheat bread fortified with bioprocessed rye bran (BRB+WW).  Nutrient content of test bread portions BRB+WW/RB+WW/R/WW (g):  Portion 166/164/123/109; protein 16/15/9/10; fat 10/15/9/7; tot DF 17/19/16/4 (tot AX 4/2/2/1, soluble AX 4/2/2/0.8, fructan 1/2/2/0.4, β-glucan 0.8/2/2/0.2). | Breads served with 40g cucumber, 20g margarine, 3 dl water, tea or coffee after an overnight fast.  Venous blood samples drawn at 0, 30, 60, 120, 180 and 240 min. | Glucose, insulin.  Repeated measures, iAUC. | Glucose:  No difference between breads.  Insulin:  iAUC was lower for the CR bread, compared to all 3 other breads.  CR bread had lower insulin response than WW at 60 min. |
| Bondia-Pons et al 2011 (81) | Cross-over, 2 test meals | 16 (13 f/3 m), age 23±3.7 y, BMI 22±1.8 kg/m^2^, normal glucose tolerance, normal blood pressure. | Commercial refined wheat bread. Commercial sourdough endosperm rye bread.  Composition of wheat bread/rye bread (g/portion): portion size 106/111, available CHO 50/50, protein 9/5, fat 5/1, DF 4/8, energy 291/230 kcal. | Bread served with 50g cucumber, 0.3g margarine and 3 dl non-caloric drink after an overnight fast.  Venous blood drawn at 0, 15, 30, 45, 60, 90, 120, 180 and 240 min. | Glucose, insulin  AUC, repeated measures | Glucose:  Rye higher than wheat bread at 90 min.  Insulin:  Rye lower than wheat at 30, 45 and 60 min. |
| Rosén et al 2011 (82) | Cross-over, 6 test meals | 14 (7 f/7 m), healthy, nonsmoking, age 21-28 y, normal BMI (mean 22.0 kg/m^2^), fasting glucose within normal range. | White endosperm wheat bread (WWB) (reference).  5 different whole grain rye breads baked with 100 % whole grain rye flour from different varieties of rye: D. Zlote (D), H. Loire (H), Nikita (N), Rekrut (R), Amilo (A).  Composition of breads WWB/D/H/N/R/A (g/serving): portion size 123/165/177169/171/172, available starch 50/50/50/50/50/50, insoluble DF 2/11/12/12/13/13, soluble DF 2/4/5/5/6/5, protein 12/13/15/13/13/13. | Breads were served with 2.5 dl water after an overnight fast.  Capillary (glucose) and venous (insulin) blood were taken at 0, 15, 30, 45, 60, 90, 120 and 180 | Glucose, insulin.  AUC_0-60_, AUC_60-120_, AUC_0-180_. Incremental glucose and insulin peak. GI and II. | Glucose:  WWB higher AUC_60-120_ than Rekrut.  Insulin:  Rekrut and Amilo had lower II than WWB, and Amilo was also lower than H. Loire.  Amilo had lower peak than all other breads, except Rekrut.  AUC_0-60_: Amilo lower than WWB, D. Zlote and Nikita.  AUC_60-120_: Amilo lower than H. Loire and WWB.  AUC_120-180_: Rekrut lower than H. Loire.  AUC_0-180_: Amilo and Rekrut lower than WWB. Rekrut lower than H. Loire. |
| Rosén, Östman and Björck 2011 (83) | Cross-over, 7 test meals | 20 (10 m/10 f), healthy, nonsmoking, age 26.7 y (range: 21-37); BMI normal range (mean 22.2 kg/m^2^); fasting glucose within normal range. | White endosperm wheat bread (WWB) (reference).  6 different whole grain rye breads baked with rye flour (3:1 whole grain rye flour:white wheat flour) from different varieties of rye: Amilo (A), Evolo (E), Kaskelott (K), Picasso (P), Vicello (V) and a commercial Swedish rye blend (C).  Composition of the breads WWB/A/E/K/P/V/C (g/serving)  Portion size: 126/155/153/154/154/149/158; water 62/73/70/71/73/69/74; protein 7/10/9/9/9/8/8; insoluble DF 2/9/9/10/10/10/11; soluble fiber 0.4/3.6/4.0/3.8/3.7/3.0/3.3; available starch 50/50/50/50/50/50/50 g. | Bread was served with 2.5 dl water, after an overnight fast.  Capillary (glucose) and venous (insulin) blood were taken at 0, 15, 30, 45, 60, 90, 120, 150 and 180 min. | Glucose, insulin  AUC_0-60_, AUC_60-120_, AUC_120-180_, AUC_0-180_.  Incremental glucose and insulin peak. GI and II. | Glucose:  Amilo, Evolo Picasso and Vicello had lower incremental peak than WWB. Picasso and Vicello had lower GI than WWB.  Amilo, Picasso, Vicello and Kaskelott had lower AUC_0-60_, compared to WWB.  Insulin:  Amilo, Evolo, Picasso and Vicello lower incremental peak, compared to WWB. Amilo, Kaskelott, Picasso and Vicello lower II compared to WWB.  Amilo, Evolo, Picasso and Vicello lower AUC_0-60_ compared to WWB.  Vicello lower AUC_0-180_ compared to WWB. |
| Rosén, Östmans and Björck 2011 (84) | Cross-over, 5 test meals | 10 (5 f/ 5 m), healthy, non-smoking, age 26.0±1.1 y; BMI 22.6±0.4 kg/m^2^. | WWB (reference): white wheat flour, water, yeast, salt. Endosperm rye bread(ERB): endosperm rye flour, water, yeast, salt.  Whole grain rye bread(WGRB): coarse whole grain rye flour, water, yeast, salt.  Rye kernels(RK): rye kernels, boiled in salted water. Wheat kernels(WK): whole grain wheat kernels, boiled in salted water.  Composition of test products WWB/ERB/WGRB/ RK/WK (g/portion)  Portion size 124/135/163/227/172; available starch 50/50/50/48/50; total starch 51/51/53/56/58 (resistant 1/1/3/8/8; protein 7/6/8/9/12; soluble DF 0.5/4.0/4.0/3.7/1.5; indigestible CHO 4/12/20/25/20; energy (kJ) 1020/1030/10801070/1170. | The bread/kernels were served after an overnight fast.  After 120 min subjects were served coffee/tea/water.  Blood samples were taken at 0, 15, 30, 45, 60, 90, 120, 180, 240, 270. Capillary blood was used for glucose analysis, venous blood was used for insulin analysis. | Glucose, insulin  AUC_0-60_, AUC_60-120_, AUC_120-270_, AUC_0-270_,  glycemic index (GI), Insulinemic index (II), repeated measures, incremental peak. | Glucose:  WGRB, RK and WK had lower GI , AUC_0-60_ min and incremental peak than WWB.  Insulin:  WWB higher II and incremental peak than all other products.  WWB had higher AUC_0-60_ and AUC_60-120_, compared to ERB, RK and WK. RK had higher AUC_120-270_, compared to WWB and WGRB. |
| Rosén et al 2009 (39) | Cross-over, 7 test meals | 12 (9 m/3 f), age 25.3±0.8 y, BMI 23.1±0.6 kg/m^2^, fasting glucose 4.6±0.03 mM, healthy. | Breads:  Wheat bread (WWB) (reference): white wheat flour.  Endosperm rye bread (ERB): Endosperm rye flour (1044g), white wheat flour (348g).  Whole grain rye bread (WGRB): whole grain rye flour (1044g), white wheat flour (348g).  Rye bran bread (RBB): white wheat flour (905g), rye bran (487g).  Porridges:  Wheat porridge (WWP): white wheat flour.  Endosperm rye porridge (ERP): endosperm rye flour (45g), white wheat flour (15g)  Whole grain rye porridge (WGRP): whole grain rye flour (51g), white wheat flour (17g).  Composition (g/serving, [mean±SEM]) (WWB/WWP/ERB/ERP/WGRB/WGRP/RBB): Portion size 101/273/106/228/123/258/142; available starch 40/38/40/38/40/39/40; protein 6/6/5/5/7/5/10; fat 2/1/1/1/2/2/3; DF 2/3/7/7/10/10/12 (insoluble DF 1/2/4/5/7/8/10, soluble DF 0.8/0.5/2.5/1.7/2.8/2.2/2.0). | Breads/porridges were served with tap water after an overnight fast. Each portion contributed 40g available starch.  Venous (for insulin) and capillary (for glucose) blood was taken at 0, 7.5, 15, 30, 45, 70, 95 and 120 min. | Glucose, insulin  iAUC_0-30_, iAUC_0-120_, GI, II, incremental insulin peak. | Glucose:  WWB had higher GI and iAUC 0-120 than ERB, ERP and WGP.  WWB had higher iAUC_0-30_ than ERB and WGB, furthermore ERB was also lower than WGRP.  Insulin:  ERB had lower peak than WWB. ERP and WGRB had lower peak than WWP. RBB had higher insulin peak than ERB, ERP and WGRB.  WWP, WGRP and RBB had higher iAUC_0-30_ than ERB and WGRB. ERB had lower iACU_0-30_ than ERP and WWB. RBB had higher iAUC_0-30_ than WGRP.  WWB and RBB had higher iAUC0-120 than ERB, ERP and WGRB. WWP had higher iAUC_0-120_ than ERB, but were lower than RBB. |
| Kallio et al 2008 (85) | Cross-over, 2 test meals | 19 (9 f/10 m), metabolic syndrome, BMI 31.9±0.7 kg/m^2^, fating plasma glucose 6.3±0.01 mmol/l, waist 109±2.1 cm. | Oat/wheat breads (reference meal): mixture of wheat bran bread, graham toast, graham crisp bread and whole meal oat bread (60% whole meal oat, 40% refined wheat)  Rye breads (rye meal): mixture 3 different commercially available whole meal rye breads and one endosperm rye bread.  Portion size (reference/rye) 125/113g; available CHO 50/50g; total DF 6/10g (soluble 2/3g); protein 16/8g; fat 5/2g; energy 1314/1031 kJ.  Half (25g) of the available CHO was from the oat bread (reference meal) and the endosperm rye bread (rye meal). | The test meals consisted of a mixture of the mentioned bread, 40g cucumber and 3 dl non-caloric beverage, and was served after an overnight fast.  Blood was drawn at 0, 15, 30, 45, 60, 90, 120, 150 and 180 min. | Glucose, insulin  Repeated measures, AUC, incremental peak | Glucose:  Rye higher than reference at 90, 120, 150 and 180 min (p<0.05).  Insulin:  Rye lower than reference at 30, 45, 60 and 90 min (p<0.05). Rye higher than reference at 150 and 180 min (p<0.05).  Rye had lower AUC (p=0.004) and lower incremental peak (p<0.001) than reference. |
| Heinonen et al 2007 (86) | Cross-over, 2 test meals | 8 (3 m/5 f), metabolic syndrome, BMI 33.7±0.7 kg/m^2^; age 55.6±1.8 y; | Rye bread: commercially available rye bread.  Control bread (reference): bread containing wheat, graham and oat grains.  Per portion (rye/control): 113/125g, 1093/1260 kJ, 50/50g available CHO, 10.2/7.0g fiber, 6.9/13.9g protein, 2.6/5.1g fat. | Breads served with 40g cucumber and 3 dl non-caloric beverage after an overnight fast. Venous blood sample drawn at 0, 15, 30, 45, 60, 90, 120 min. | Glucose, insulin  Repeated measures and AUC | Glucose:  No difference  Insulin:  Rye lower AUC than wheat.  Rye lower than wheat at 30 and 60 minutes. |
| Juntunen, Laaksonen Autio et al 2003 (56) | Cross-over, 4 test meals | 19 women, healthy, age 61±4.8 (range: 51-69) y; BMI 26.0±2.5 (22.5-30.2) kg/m^2^ | Refined wheat bread (RW) (reference): content not reported  Endosperm rye bread (ER): endosperm rye flour, sourdough (endosperm rye flour), water, yeast, salt.  Traditional rye bread (TR): whole meal rye flour, sourdough (whole meal rye flour), water, yeast, salt.  High-fiber rye bread (HR): Whole meal rye flour, rye bran, sourdough (whole meal rye flour), water, yeast, salt.  Per portion (RW/ER/TR/HR): portion size 106/112/143/199g; available CHO 50g; total DF 3/6/15/29g (insoluble 1/3/11/24g, soluble 1/3/4/5g); protein 9/5/11/17g; fat 5/3/8/10g; energy 1177/1056/1295/1486 kJ. | Breads served with 40g cucumber and 3 dl non-caloric beverage after an overnight fast. Refined wheat served at 2 separate occasions, and the mean was used for statistical analysis.  Venous blood samples drawn at 0, 15, 30, 45, 60, 90, 120, 150 and 180 min. | Glucose, insulin  Repeated measures, incremental peak values, AUC | Glucose:  No difference in glucose.  Insulin:  All rye breads had lower incremental peak value than RW.  ER and TR had lower AUC than RW.  All 3 rye breads were lower than RW at 45, 60 and 90 min. ER was lower than RW at 30 min, but higher at 180 min. HR was higher than RW at 180 min. |
| Juntunen et al 2002 (87) | Cross-over, partially randomized (BR bread was served at last occasion for all subjects),  2 test meals | 20 (10 m/10 f), healthy, normal glucose tolerance, age (y): 29±1.8 (m), 28±1.8 (f), BMI: (kg/m2) 24.3±1.0 (m), 21.5±0.6 (f), | White wheat bread (WW) (reference): White wheat flour, water, yeast, salt, margarine, paracetamol, bread improver.  Whole-kernel rye bread (WKR): Rye kernels, sifted rye flour, whole meal rye flour, yeast, salt, paracetamol, lactic acid.  Nutrient composition of WW/WKR (g/portion):  Portion size: 112.4/135; available CHO 50/50; tot DF 3/13 (soluble 0.9/4, pentosan 2/9, β-glucan 0.2/1); protein 8/7; fat 3/3; energy (kJ) 1117/1084. | Breads served with 40g cucumber and 3 dl non-caloric beverage after an overnight fast. Pasta served with 19g crushed tomatoes and 3 dl non-caloric beverage.  Refined wheat served at 2 separate occasions, and the mean was used for statistical analysis.  Venous blood samples drawn at 0, 15, 30, 45, 60, 90, 120, 150 and 180 min. | Glucose, insulin  Incremental peak and repeated measures (0-180 min) | Glucose:  WW had higher glucose peak than WP.  Insulin:  WKR lower than WW at 30, 45, 90, 120 and 150 min.  WW had higher insulin peak than WKR. |
| Leinonen et al 1999 (88) | Cross-over, 4 test meals | 20 (10 m/10 f), healthy with normal glucose tolerance, age (y): 32±3 (m), 27±5 (f); BMI 24.5±2.2 (m), 20.3±1.1 (f). | Whole kernel rye bread (WKR).  White wheat bread (WW) (reference). All breads were commercially available, finely milled rye flour used in rye breads.  Nutrient composition of test bread portions WW/WKR (g)  Portion size 121/148; available CHO 61/55 (free sugars 3/7); DF 2/14; protein 11/9; fat 3/2; energy (kJ) 1341/1173. | Breads served after an overnight fast.  Venous blood samples drawn at 0, 15, 30, 45, 60, 90, 120, 150 and 180 min. | Glucose and insulin  Repeated measures, AUC, incremental peak. | Glucose: No difference.  Insulin: WW higher at 45, 60, 90, 120, and 150 min. AUC lower for WKR. Incremental peak lower for WKR than for WW. |
| Liljeberg and Björck et al 1994 (89) | Cross-over, 2 test meals | 8 (5 m/3 f), age 25-47 y, normal BMI, healthy. | Wheat bread (WWB): White wheat flour, water, yeast.  Barley kernel bread (SCB80): 80% scalded barley kernels, whole meal barley flour, white wheat flour, sourdough.  Barley kernel bread (SCB40): 40% scalded barley kernels, whole meal barley flour, white wheat flour, sourdough.  Whole meal barley bread ( WMB): Whole meal barley flour, white wheat flour, yeast.  Whole meal barley sourdough bread (WMBS): Whole meal barley flour, white wheat flour, sourdough.  Scaled whole meal barley bread (SWMB): Scalded whole meal barley flour, white wheat flour, yeast.  Pumpernickel (PB): Rye kernels, white wheat flour, rye malt, yeast (commercial product).  Composition (g/100g) WWB/SCB80/SCB40/WMB/WMBS/SWMB/PB: starch 79/65/69/66/65/65/56, protein 12/12/12/12/12/12/9, fat 3/4/4/4/4/4/4, DF 4/15/10/11/13/13/14 (insoluble DF 2/9/6/6/8/8/9, soluble DF 4/6/4/5/5/5/14). | Bread served with cheese, butter and water/tea/coffee, after an overnight fast. Each serving had 50 g starch.  Blood drawn at:  0, 30, 45, 70, 95, 120 and 180 (glucose, capillary blood)  0, 30, 45, 95 and 120 (insulin) | Glucose, insulin  GI, II AUC. | Glucose:  PB lower AUC and GI than WWB, but higher than SC80  Insulin:  PB lower AUC and II than WWB. |
| Elliot et al 1994 (90) | Cross-over, 2 test meals | 6, healthy, 19-23 y, BMI 20-35 kg/m^2^. | Wheat bread (reference): commercial whole meal wheat bread. Rye bread: commercial Italian style rye bread (50% rye). Breads were chosen to have similar processing and composition.  Nutritional composition wheat/rye (g/portion):  Portion size 120/126; energy 1135/1071 kJ; CHO 50/50; protein 12/10; fat 3/2; fiber 9/9. | Bread served with 2.5 dl skimmed milk, after an overnight fast.  Venous blood drawn at -15, 0, 15 30, 45, 60, 90, 120, 150 and 180 min. | Glucose, insulin  Repeated measures, AUC | Glucose:  No difference  Insulin:  No difference |
| Liljeberg, Granfeldt and Björck 1992 (73) | Cross-over, 3 test meals | 10 (5 m/5 f), age 36-50 y, normal BMI, healthy. | White wheat bread (WWB) (reference): 100% white wheat flour.  Coarse wheat bread (CWB): 80% intact wheat kernels, 20 white wheat flour.  Coarse rye bread (CRB): 80% boiled intact rye kernels, 20% white wheat flour.  Coarse oat bread (COB): 80% intact oat kernels, 20% white wheat flour.  Whole meal barley bread (WMBB): 83% whole meal barley flour, 17% white wheat flour.  Coarse barley bread (CBB): 80% intact barley kernels, 20% white wheat flour.  Scalded coarse barley bread (SCBB): 80% intact scalded barley kernels, 20% white wheat.  Composition (g/100g dry weight) WWB/CWB/CRB/COB/WMBB/CBB/SCBB: starch 78/66/63/64/65/65/65, protein 13/13/11/14/14/14/14, fat 3/4/4/8/4/4/4, DF 3/10/13/8/10/10/10 (insoluble DF 2/9/9/6/6/7/7, soluble DF 1/1/4//3/4/3/3).  Portion size: 103/143/146/159/147/156/147g. | Bread served with cheese, butter and water/tea/coffee, after an overnight fast. Each serving had 50 g starch.  Blood drawn at:  0, 30, 45, 70, 95, 120 and 180 (glucose, capillary blood)  0, 30, 45, 95 and 120 (insulin) | Glucose, insulin  GI, II, AUC | Glucose:  CRB had lower AUC than WWB and WMBB.WWB, COB and WMBB had higher Gi than CRB.  Insulin:  CRB had lower AUC than CWB, COB and WMRB. WWB, CWB, OCB and WMBB had higher II than CRB. |
| Hagander et al 1987 (91) | Cross-over, 3 test meals | 7 (4 f/3 m), non-insulin dependent diabetics, age 56-73 y, BMI (kg/m^2^) 31.7±2.3 (f), 27.9±1.4. | Wheat bread (reference): White wheat flour, yeast fermented (serving size 92g).  Rye bread: whole grain rye flour (2290g), white wheat flour (925g) , yeast fermented (serving size 113g).  Rye flakes: rolled rye flakes, 55g/portion (served with one slice wheat bread (30g) to match rye/wheat ratio of rye bread).  Composition of test meals, wheat bread/rye bread/rye flakes (g/portion): starch 5/5/5, fat 18/18/18, protein 18/18/18, DF 3/10/9 (soluble DF 1/2/2), energy 468/463/465 kcal. | Meals consisted of test product, margarine, cheese, coffee, water and yogurt. One subject had milk instead of yoghurt. Meals served after an overnight fast.  Continuous glucose monitoring over 3 hours (0-180 min). Venous blood for insulin at 0, 10, 20, 30, 45, 60, 90, 120, 150 and 180 min. | Glucose: AUC, delay time, rate of rise, plateau level, rate of decrease.  Insulin: AUC, repeated measures | Glucose:  Rye bread had lower AUC than wheat bread. Wheat bread had higher rate of decrease than rye flakes. Rye bread had lower rate of rise than rye flakes.  Insulin:  Rye flakes higher than rye bread at 150 min. |

^ǂ^Data is mean±sd or (range), *Only test meals and outcomes of interest for investigating the rye factor (insulin, glucose) is included in the table. Abbreviations: AUC, area under the curve; BMI, body mass index; CHO, carbohydrate; DF, dietary fiber; DM, dry matter; f, female; GI, glycemic index; iAUC, incremental area under the curve; II, insulinemic index; m, male; na, not available; y, year
